# *catena*-Poly[[triaqua­nickel(II)]-μ-5-carb­oxy­benzene-1,3-dicarboxyl­ato-κ^2^ *O* ^1^:*O* ^3^]

**DOI:** 10.1107/S1600536811035227

**Published:** 2011-09-14

**Authors:** Xing-Jun Yao, Qian Yuan

**Affiliations:** a College of Chemistry and Chemical Engineering, Liaocheng University, 252059 Liaocheng, Shandong, People’s Republic of China; b Guodian Liaocheng Power Co. Ltd, 252033 Liaocheng, Shandong, People’s Republic of China

## Abstract

In the title compound, [Ni(C_9_H_4_O_6_)(H_2_O)_3_]_*n*_, the Ni^II^ ion has a distorted NiO_5_ square-pyramidal geometry, the maximum deviation from the least-squares plane formed by the basal atoms being 0.9351 (13) Å. The basal plane is formed by two O atoms from carboxyl­ate residues of the 5-carb­oxy­benzene-1,3-dicarboxyl­ate ligand and by two O atoms from water mol­ecules. The O atom of the third water mol­ecule is axially positioned, 1.7890 (19) Å perpendicular to the basal plane. The 5-carb­oxy­benzene-1,3-dicarboxyl­ate ligand bridges the metal atoms, forming a polymeric chain along the *b* axis. O—H⋯O hydrogen bonds between the water mol­ecules and carboxyl­ate groups stabilize the crystal structure.

## Related literature

For the applications and stuctures of related metal complexes of 1,3,5-benzene­tricarb­oxy­lic acid, see: Xia *et al.* (2004 ▶); Modec & Brencic (2005 ▶); Wei & Han (2005 ▶); Han & Wei (2005 ▶); Wang *et al.* (2005 ▶); Che *et al.* (2008 ▶); He *et al.* (2008 ▶); Li *et al.* (2008 ▶); Gao *et al.* (2009 ▶). For bond-length data, see: Allen *et al.* (1987 ▶).
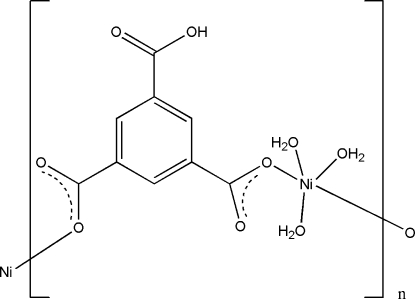


## Experimental

### 

#### Crystal data

[Ni(C_9_H_4_O_6_)(H_2_O)_3_]
*M* *_r_* = 320.88
Monoclinic, 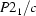

*a* = 6.838 (2) Å
*b* = 18.809 (5) Å
*c* = 10.705 (3) Å
β = 126.901 (14)°
*V* = 1101.0 (5) Å^3^
*Z* = 4
Mo *K*α radiation
μ = 1.81 mm^−1^
*T* = 296 K
0.30 × 0.25 × 0.20 mm

#### Data collection

Bruker SMART APEXII CCD area-detector diffractometer
Absorption correction: multi-scan (*SADABS*; Bruker, 2005 ▶) *T*_min_ = 0.613, *T*_max_ = 0.714
7973 measured reflections
1938 independent reflections
1864 reflections with *I* > 2σ(*I*)
*R*_int_ = 0.021

#### Refinement

*R*[*F* ^2^ > 2σ(*F* ^2^)] = 0.023
*wR*(*F* ^2^) = 0.102
*S* = 1.01
1938 reflections
173 parameters
H-atom parameters constrained
Δρ_max_ = 0.32 e Å^−3^
Δρ_min_ = −0.38 e Å^−3^

### 

Data collection: *APEX2* (Bruker, 2005 ▶); cell refinement: *SAINT* (Bruker, 2005 ▶); data reduction: *SAINT*; program(s) used to solve structure: *SHELXTL* (Sheldrick, 2008 ▶); program(s) used to refine structure: *SHELXTL*; molecular graphics: *SHELXTL*; software used to prepare material for publication: *SHELXTL*.

## Supplementary Material

Crystal structure: contains datablock(s) global, I. DOI: 10.1107/S1600536811035227/go2022sup1.cif

Structure factors: contains datablock(s) I. DOI: 10.1107/S1600536811035227/go2022Isup2.hkl

Additional supplementary materials:  crystallographic information; 3D view; checkCIF report

## Figures and Tables

**Table 1:** Selected bond lengths (Å)

Ni1—O1	1.9292 (14)
Ni1—O2*W*	1.9781 (17)
Ni1—O1*W*	1.9884 (18)
Ni1—O3*W*	2.2536 (16)
O6—Ni1^i^	1.9129 (15)

Symmetry code: (i) 
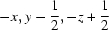
.

**Table 2:** Hydrogen-bond geometry (Å, °)

*D*—H⋯*A*	*D*—H	H⋯*A*	*D*⋯*A*	*D*—H⋯*A*
O3—H3*A*⋯O2^ii^	0.82	1.81	2.568 (2)	152
O1*W*—H1*W*⋯O3^iii^	0.85	2.21	2.869 (3)	134
O1*W*—H2*W*⋯O5^iv^	0.85	1.94	2.680 (2)	145
O2*W*—H4*W*⋯O5^v^	0.85	1.89	2.715 (2)	165
O3*W*—H5*W*⋯O4^vi^	0.85	2.03	2.778 (2)	147
O3*W*—H6*W*⋯O2^vii^	0.85	2.49	3.068 (3)	126
O2*W*—H3*W*⋯O1^viii^	0.85	2.34	3.123 (2)	154

Symmetry codes: (ii) 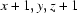; (iii) 
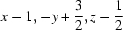
; (iv) 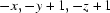; (v) 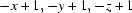; (vi) 
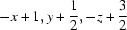
; (vii) 
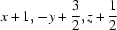
; (viii) 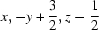.

**Fig. 1.**
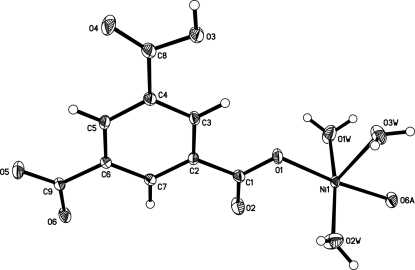
The molecular structure of (I), with displacement ellipsoids drawn at the 30% probability level [symmetry codes: (A) -x, 1/2 + y, 1/2 - z]

**Fig. 2.**
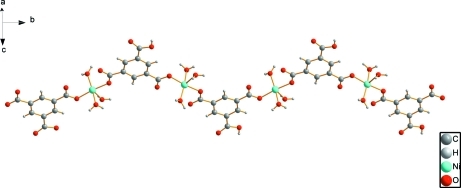
View of the chain structure in the title compound.

## supplementary crystallographic information

### Comment

In recent years, the construction of metal complexes based on 1,3,5-benzenetricarboxylic acid ligand has been investigated owing to their potential applications in many fields (Xia *et al.*, 2004; Modec & Brencic, 2005; Wei & Han, 2005; Han & Wei, 2005; Wang *et al.*, 2005; Che *et al.*, 2008; He *et al.*, 2008; Li *et al.*, 2008; Gao *et al.*, 2009). In order to search for new metal complex based on 1,3,5-benzenetricarboxylic acid ligand, the title complex, (I) was synthesized and its crystal determined (Fig. 1). The bond lengths and angles are normal (Allen *et al.*, 1987). In the crystal structure, the HBTC ligands bridge the Ni atoms, forming a chain along the *b* axis (Fig. 2). O—H···O hydrogen bonds between the water molecules and carboxylate groups stabilize the structure.

### Experimental

A mixture of NiNO_3_^.^6H_2_O (0.10 mmol), 1,3,5-benzenetricarboxylic acid (H3BTC, 0.10 mmol), Et_3_N (0.1 ml), EtOH (2 ml) and H_2_O (2 ml) was sealed in a 10 ml Tefon-lined stainless-steel reactor and then heated to 393 K for 48 h under autogenous pressure. The mixture was slowly cooled to room temperature. Green block crystals suitable for X-ray diffraction analysis were collected by filtration..

### Refinement

H atoms attached to C atoms were placed in calculated positions (C—H = 0.93 Å) and refined as riding atoms and with *U*_iso_(H) = 1.2 *U*_eq_(C, N),respectively. The carboxy and water H atoms were located in a difference map and refined with O—H bond length from 0.82 to 0.85 Å and *U*_iso_(H) = 1.5 *U*_eq_(O).

### Crystal data

**Table:**

[Ni(C_9_H_4_O_6_)(H_2_O)_3_]	*F*(000) = 656
*M**_r_* = 320.88	*D*_x_ = 1.936 Mg m^−^^3^
Monoclinic, *P*2_1_/*c*	Mo *K*α radiation, λ = 0.71073 Å
Hall symbol: -P 2ybc	Cell parameters from 6807 reflections
*a* = 6.838 (2) Å	θ = 2.6–27.8°
*b* = 18.809 (5) Å	µ = 1.81 mm^−^^1^
*c* = 10.705 (3) Å	*T* = 296 K
β = 126.901 (14)°	Block, green
*V* = 1101.0 (5) Å^3^	0.30 × 0.25 × 0.20 mm
*Z* = 4	

### Data collection

**Table:**

Bruker SMART APEXII CCD area-detector diffractometer	1938 independent reflections
Radiation source: fine-focus sealed tube	1864 reflections with *I* > 2σ(*I*)
graphite	*R*_int_ = 0.021
φ and ω scans	θ_max_ = 25.0°, θ_min_ = 2.2°
Absorption correction: multi-scan (*SADABS*; Bruker, 2005)	*h* = −8→8
*T*_min_ = 0.613, *T*_max_ = 0.714	*k* = −22→21
7973 measured reflections	*l* = −10→12

### Refinement

**Table:**

Refinement on *F*^2^	Secondary atom site location: difference Fourier map
Least-squares matrix: full	Hydrogen site location: inferred from neighbouring sites
*R*[*F*^2^ > 2σ(*F*^2^)] = 0.023	H-atom parameters constrained
*wR*(*F*^2^) = 0.102	*w* = 1/[σ^2^(*F*_o_^2^) + (0.0942*P*)^2^ + 0.090*P*] where *P* = (*F*_o_^2^ + 2*F*_c_^2^)/3
*S* = 1.01	(Δ/σ)_max_ = 0.002
1938 reflections	Δρ_max_ = 0.32 e Å^−^^3^
173 parameters	Δρ_min_ = −0.38 e Å^−^^3^
0 restraints	Extinction correction: *SHELXTL* (Sheldrick, 2008), Fc^*^=kFc[1+0.001xFc^2^λ^3^/sin(2θ)]^-1/4^
Primary atom site location: structure-invariant direct methods	Extinction coefficient: 0.036 (3)

### Special details

**Table:**

Geometry. All e.s.d.'s (except the e.s.d. in the dihedral angle between two l.s. planes)are estimated using the full covariance matrix. The cell e.s.d.'s are takeninto account individually in the estimation of e.s.d.'s in distances, anglesand torsion angles; correlations between e.s.d.'s in cell parameters are onlyused when they are defined by crystal symmetry. An approximate (isotropic)treatment of cell e.s.d.'s is used for estimating e.s.d.'s involving l.s. planes.
Refinement. Refinement of *F*^2^ against ALL reflections. The weighted *R*-factor *wR* andgoodness of fit *S* are based on *F*^2^, conventional *R*-factors *R* are basedon *F*, with *F* set to zero for negative *F*^2^. The threshold expression of*F*^2^ > σ(*F*^2^) is used only for calculating *R*-factors(gt) *etc*. and isnot relevant to the choice of reflections for refinement. *R*-factors basedon *F*^2^ are statistically about twice as large as those based on *F*, and *R*-factors based on ALL data will be even larger.

### Fractional atomic coordinates and isotropic or equivalent isotropic displacement parameters (Å^2^)

**Table:**

	*x*	*y*	*z*	*U*_iso_*/*U*_eq_	
C1	0.1199 (3)	0.63000 (10)	0.3748 (2)	0.0228 (4)	
C2	0.2227 (3)	0.56454 (10)	0.4747 (2)	0.0203 (4)	
C3	0.3935 (3)	0.57173 (10)	0.6364 (2)	0.0213 (4)	
H3	0.4431	0.6168	0.6806	0.026*	
C4	0.4892 (3)	0.51227 (11)	0.7312 (2)	0.0220 (4)	
C5	0.4154 (3)	0.44487 (11)	0.6640 (2)	0.0231 (4)	
H5	0.4805	0.4047	0.7273	0.028*	
C6	0.2465 (3)	0.43711 (10)	0.5040 (2)	0.0196 (4)	
C7	0.1480 (3)	0.49745 (10)	0.4085 (2)	0.0209 (4)	
H7	0.0327	0.4925	0.3010	0.025*	
C8	0.6645 (4)	0.51904 (11)	0.9031 (2)	0.0281 (5)	
C9	0.1625 (3)	0.36441 (10)	0.4346 (2)	0.0214 (4)	
Ni1	0.11091 (4)	0.773616 (12)	0.32089 (3)	0.01894 (19)	
O1	0.2116 (3)	0.68864 (7)	0.44634 (16)	0.0274 (4)	
O2	−0.0451 (3)	0.62603 (8)	0.23299 (18)	0.0408 (4)	
O3	0.7481 (3)	0.58432 (8)	0.95119 (18)	0.0371 (4)	
H3A	0.8391	0.5853	1.0470	0.056*	
O4	0.7292 (4)	0.47053 (9)	0.99436 (19)	0.0532 (6)	
O5	0.2577 (3)	0.31100 (8)	0.51934 (18)	0.0314 (4)	
O6	−0.0066 (3)	0.36214 (7)	0.28913 (17)	0.0304 (4)	
O1W	−0.1375 (4)	0.78863 (9)	0.3587 (2)	0.0448 (5)	
H1W	−0.0823	0.8266	0.4123	0.067*	
H2W	−0.1097	0.7563	0.4230	0.067*	
O2W	0.3070 (3)	0.74742 (11)	0.2478 (2)	0.0430 (4)	
H4W	0.4388	0.7241	0.3076	0.065*	
H3W	0.3268	0.7712	0.1886	0.065*	
O3W	0.4158 (3)	0.82950 (8)	0.53994 (18)	0.0394 (4)	
H5W	0.4312	0.8744	0.5432	0.059*	
H6W	0.5507	0.8132	0.5647	0.059*	

### Atomic displacement parameters (Å^2^)

**Table:**

	*U*^11^	*U*^22^	*U*^33^	*U*^12^	*U*^13^	*U*^23^
C1	0.0263 (9)	0.0193 (10)	0.0166 (9)	0.0008 (7)	0.0096 (7)	−0.0008 (8)
C2	0.0241 (9)	0.0153 (10)	0.0193 (9)	−0.0015 (7)	0.0119 (8)	0.0002 (7)
C3	0.0246 (9)	0.0140 (10)	0.0209 (9)	−0.0032 (7)	0.0113 (8)	−0.0021 (8)
C4	0.0240 (10)	0.0193 (9)	0.0181 (10)	−0.0005 (8)	0.0103 (8)	−0.0008 (8)
C5	0.0285 (9)	0.0155 (10)	0.0227 (10)	0.0014 (7)	0.0141 (8)	0.0032 (7)
C6	0.0238 (9)	0.0163 (10)	0.0192 (9)	−0.0016 (7)	0.0132 (8)	−0.0018 (7)
C7	0.0239 (9)	0.0194 (10)	0.0181 (9)	−0.0020 (8)	0.0119 (8)	−0.0025 (8)
C8	0.0316 (10)	0.0233 (11)	0.0215 (10)	−0.0010 (8)	0.0117 (9)	−0.0021 (8)
C9	0.0246 (9)	0.0170 (10)	0.0243 (10)	−0.0003 (7)	0.0157 (8)	−0.0014 (8)
Ni1	0.0249 (2)	0.0112 (3)	0.0160 (3)	0.00245 (7)	0.00978 (19)	0.00329 (7)
O1	0.0346 (8)	0.0141 (7)	0.0212 (7)	−0.0010 (5)	0.0102 (6)	0.0009 (6)
O2	0.0520 (10)	0.0239 (9)	0.0193 (8)	0.0012 (7)	0.0070 (7)	0.0010 (7)
O3	0.0508 (10)	0.0231 (8)	0.0189 (8)	−0.0080 (7)	0.0111 (7)	−0.0034 (6)
O4	0.0761 (13)	0.0245 (9)	0.0189 (8)	−0.0031 (8)	0.0072 (8)	0.0062 (7)
O5	0.0348 (8)	0.0179 (8)	0.0331 (8)	−0.0015 (6)	0.0159 (7)	0.0024 (6)
O6	0.0358 (8)	0.0196 (7)	0.0222 (7)	−0.0037 (6)	0.0102 (6)	−0.0057 (6)
O1W	0.0583 (11)	0.0283 (8)	0.0682 (13)	0.0123 (9)	0.0489 (11)	0.0156 (9)
O2W	0.0453 (10)	0.0524 (11)	0.0384 (9)	0.0201 (9)	0.0289 (9)	0.0161 (9)
O3W	0.0399 (8)	0.0279 (9)	0.0331 (8)	−0.0027 (7)	0.0126 (7)	−0.0066 (7)

### Geometric parameters (Å, °)

**Table:**

C1—O2	1.236 (3)	C9—O5	1.244 (2)
C1—O1	1.273 (2)	C9—O6	1.266 (3)
C1—C2	1.500 (3)	Ni1—O6^i^	1.9129 (15)
C2—C7	1.386 (3)	Ni1—O1	1.9292 (14)
C2—C3	1.397 (3)	Ni1—O2W	1.9781 (17)
C3—C4	1.383 (3)	Ni1—O1W	1.9884 (18)
C3—H3	0.9300	Ni1—O3W	2.2536 (16)
C4—C5	1.394 (3)	O3—H3A	0.8200
C4—C8	1.480 (3)	O6—Ni1^ii^	1.9129 (15)
C5—C6	1.383 (3)	O1W—H1W	0.8501
C5—H5	0.9300	O1W—H2W	0.8500
C6—C7	1.400 (3)	O2W—H4W	0.8501
C6—C9	1.496 (3)	O2W—H3W	0.8500
C7—H7	0.9300	O3W—H5W	0.8501
C8—O4	1.210 (3)	O3W—H6W	0.8500
C8—O3	1.321 (3)		
			
O2—C1—O1	123.25 (18)	O5—C9—C6	119.96 (17)
O2—C1—C2	121.17 (17)	O6—C9—C6	115.87 (16)
O1—C1—C2	115.58 (16)	O6^i^—Ni1—O1	174.25 (7)
C7—C2—C3	119.92 (18)	O6^i^—Ni1—O2W	93.67 (7)
C7—C2—C1	120.79 (17)	O1—Ni1—O2W	91.48 (7)
C3—C2—C1	119.28 (17)	O6^i^—Ni1—O1W	87.32 (7)
C4—C3—C2	120.44 (18)	O1—Ni1—O1W	88.13 (7)
C4—C3—H3	119.8	O2W—Ni1—O1W	168.69 (8)
C2—C3—H3	119.8	O6^i^—Ni1—O3W	90.27 (6)
C3—C4—C5	119.43 (18)	O1—Ni1—O3W	86.65 (6)
C3—C4—C8	121.08 (18)	O2W—Ni1—O3W	96.01 (8)
C5—C4—C8	119.48 (17)	O1W—Ni1—O3W	95.25 (8)
C6—C5—C4	120.64 (18)	C1—O1—Ni1	117.27 (12)
C6—C5—H5	119.7	C8—O3—H3A	109.5
C4—C5—H5	119.7	C9—O6—Ni1^ii^	120.96 (13)
C5—C6—C7	119.76 (18)	Ni1—O1W—H1W	99.8
C5—C6—C9	119.82 (18)	Ni1—O1W—H2W	105.1
C7—C6—C9	120.37 (16)	H1W—O1W—H2W	105.1
C2—C7—C6	119.80 (17)	Ni1—O2W—H4W	119.7
C2—C7—H7	120.1	Ni1—O2W—H3W	127.8
C6—C7—H7	120.1	H4W—O2W—H3W	105.1
O4—C8—O3	121.55 (19)	Ni1—O3W—H5W	121.4
O4—C8—C4	124.72 (19)	Ni1—O3W—H6W	108.0
O3—C8—C4	113.73 (18)	H5W—O3W—H6W	105.1
O5—C9—O6	124.16 (18)		
			
O2—C1—C2—C7	4.9 (3)	C3—C4—C8—O4	169.1 (2)
O1—C1—C2—C7	−176.04 (17)	C5—C4—C8—O4	−9.6 (3)
O2—C1—C2—C3	−174.3 (2)	C3—C4—C8—O3	−10.9 (3)
O1—C1—C2—C3	4.8 (3)	C5—C4—C8—O3	170.43 (19)
C7—C2—C3—C4	0.2 (3)	C5—C6—C9—O5	−5.2 (3)
C1—C2—C3—C4	179.35 (17)	C7—C6—C9—O5	177.22 (18)
C2—C3—C4—C5	0.6 (3)	C5—C6—C9—O6	174.46 (18)
C2—C3—C4—C8	−178.15 (18)	C7—C6—C9—O6	−3.1 (3)
C3—C4—C5—C6	−0.6 (3)	O2—C1—O1—Ni1	−7.3 (3)
C8—C4—C5—C6	178.15 (19)	C2—C1—O1—Ni1	173.69 (13)
C4—C5—C6—C7	−0.1 (3)	O2W—Ni1—O1—C1	−72.99 (16)
C4—C5—C6—C9	−177.68 (17)	O1W—Ni1—O1—C1	95.70 (16)
C3—C2—C7—C6	−0.8 (3)	O3W—Ni1—O1—C1	−168.93 (15)
C1—C2—C7—C6	179.97 (17)	O5—C9—O6—Ni1^ii^	−6.8 (3)
C5—C6—C7—C2	0.8 (3)	C6—C9—O6—Ni1^ii^	173.52 (12)
C9—C6—C7—C2	178.38 (17)		

Symmetry codes: (i) −*x*, *y*+1/2, −*z*+1/2; (ii) −*x*, *y*−1/2, −*z*+1/2.

### Hydrogen-bond geometry (Å, °)

**Table:**

*D*—H···*A*	*D*—H	H···*A*	*D*···*A*	*D*—H···*A*
O3—H3A···O2^iii^	0.82	1.81	2.568 (2)	152.
O1W—H1W···O3^iv^	0.85	2.21	2.869 (3)	134.
O1W—H2W···O5^v^	0.85	1.94	2.680 (2)	145.
O2W—H4W···O5^vi^	0.85	1.89	2.715 (2)	165.
O3W—H5W···O4^vii^	0.85	2.03	2.778 (2)	147.
O3W—H6W···O2^viii^	0.85	2.49	3.068 (3)	126.
O2W—H3W···O1^ix^	0.85	2.34	3.123 (2)	154.

Symmetry codes: (iii) *x*+1, *y*, *z*+1; (iv) *x*−1, −*y*+3/2, *z*−1/2; (v) −*x*, −*y*+1, −*z*+1; (vi) −*x*+1, −*y*+1, −*z*+1; (vii) −*x*+1, *y*+1/2, −*z*+3/2; (viii) *x*+1, −*y*+3/2, *z*+1/2; (ix) *x*, −*y*+3/2, *z*−1/2.
